# Wnt7a induces satellite cell expansion, myofiber hyperplasia and hypertrophy in rat craniofacial muscle

**DOI:** 10.1038/s41598-018-28917-6

**Published:** 2018-07-13

**Authors:** Xu Cheng, Hanyao Huang, Xiangyou Luo, Bing Shi, Jingtao Li

**Affiliations:** State Key Laboratory of Oral Diseases & National Clinical Research Centre for Oral Diseases & Department of Oral and Maxillofacial Surgery, West China Hospital of Stomatology, Sichuan University, 14 Ren Min Nan Road, Chengdu, 610041 P. R. China

## Abstract

Craniofacial muscles drive critical functions in the head, including speech, feeding and expression. Compared with their counterparts in trunk and limbs, craniofacial muscles are of distinct embryonic origins, which might consequently lead to different growth patterns and regenerative potential. In this study, rat levator veli palatini muscle and masseter muscle were compared with tibialis anterior muscle in their response to exogenous Wnt7a stimulus, which has been proved effective in promoting muscle regeneration in the limbs. Histological, cellular and molecular analyses were performed both under basal condition and after a single dose injection of recombinant human Wnt7a. Under basal condition, levator veli palatini muscle demonstrated considerably more satellite cells than the others. After Wnt7a administration, regeneration-related activities, including satellite cell expansion, myofiber hyperplasia and hypertrophy were generally observed in all three muscles, but with obvious differences in the extent. The composition of fast/slow myofibers underwent substantial alterations, and the pattern varied among the three muscles. Location-specific alterations in the expression level of core components in planar cell polarity pathway, Akt/mTOR pathway and myostatin pathway were also observed. In conclusion, both craniofacial and limb muscles could be effectively expanded by exogenous Wnt7a stimulus, but muscle-to-muscle variations in response patterns existed.

## Introduction

Skeletal muscle is highly coordinated and malleable tissue and compromises up to 40% of the total body mass^1^. With intrinsic properties to withstand stress and produce force, skeletal muscle supports body posture and drives body movements, ranging from sudden and intensive limb movements like jumping and kicking, to continuous and mild activities like respiration, speech and expressions in the craniofacial region^2^. These critical functions could be impaired when pathological alterations occur to skeletal muscle.

The most studied skeletal muscle degenerative disease is Duchenne muscular dystrophy, which drags in extensive trunk and limb muscles, incurring delayed walking and repeated falls^3^. While in the craniofacial region, skeletal muscle insufficiency or incompetence lead to equally troublesome dysfunctions. For example, levator veli palatini (LVP) muscle contracts to pull the soft palate upwards, so as to achieve complete separation between oral and nasal cavities and thus form normal speech^4^. Atrophic changes of LVP muscle in congenital cleft palate deformity^4–6^, result in up to 30% of post-operative cleft palate patients suffering ambiguous speech^7,8^. Likewise, in hemifacial microsomia, the masseter (MAS) muscle demonstrates hypoplastic alterations, compromising both facial aesthetics and occlusal function^9^. Thus, effective therapeutic for muscle regeneration is in desperate need for not only limb and trunk muscles, but also craniofacial muscles.

Muscle regeneration studies, however, have been focused on limb muscles, leaving a paucity of data considering craniofacial muscle regeneration. Limb muscles derive from somites, but the majority of craniofacial muscles develop from branchial arches^10^. Limb muscles were demonstrated to possess a smaller population of muscle stem cells than craniofacial muscles^11^. In addition, data from limb muscle studies is only valuable for illustrating clinical problems in tailored settings^1,3^: Duchenne muscular dystrophy preferentially inflicted on limb muscles with craniofacial muscles spared. In light of these established differences between craniofacial muscle and limb muscle, therapeutic possibilities to regenerate limb muscle need to be retested on craniofacial muscle.

The efforts searching for cures to regenerate limb muscle have been undertaken for decades, and multiple growth factors have been suggested of therapeutic potentials. IGF-1 delivery could promote muscle stem cell activation and terminal differentiation^12^. VEGF administration could increase myofiber diameter and the number of centrally located nuclei^13^. Recent studies on limb muscle regeneration have revealed that, a novel Wnt ligand, Wnt7a, was able to ameliorate muscular dystrophy symptoms both in mice and humans, with the merit of avoiding hypoglycaemia, the common complication occurring in other growth factor administration^14^. Successful delivery of Wnt7a to targeted muscles could regulate Wnt signalling pathway and intervene downstream reactions to correct the pathological consequences. Specifically, recombinant human Wnt7a (rh-Wnt7a) administration could activate the planar cell polarity (PCP) pathways to expand muscle stem cell population^15^ as well as mediating Akt/mTOR pathway to induce myofiber hypertrophy^14^.

With the heterogeneity between limb muscles and craniofacial muscles in mind, this study set out to interrogate both the effectiveness and mechanism of rh-Wnt7a on craniofacial muscles. In a rat model, the potential regenerative responses of craniofacial LVP muscle and MAS muscle were illustrated in details, using the limb tibialis anterior (TA) muscle as a control.

## Results

### Regeneration-related activities vary among LVP, MAS and TA muscle

Our previous study demonstrated disparate histological and cellular phenotypes between limb and craniofacial muscles^16^. At the beginning of this study we set out to examine the stem cell population, proliferation, and regenerative activity among the fourth branchial arch-derived LVP muscle^17^, the first branchial arch-derived MAS muscle^17^ and the somite-derived TA muscle^18^ in rats, both at three weeks and ten weeks after birth.

**Figure 1:**
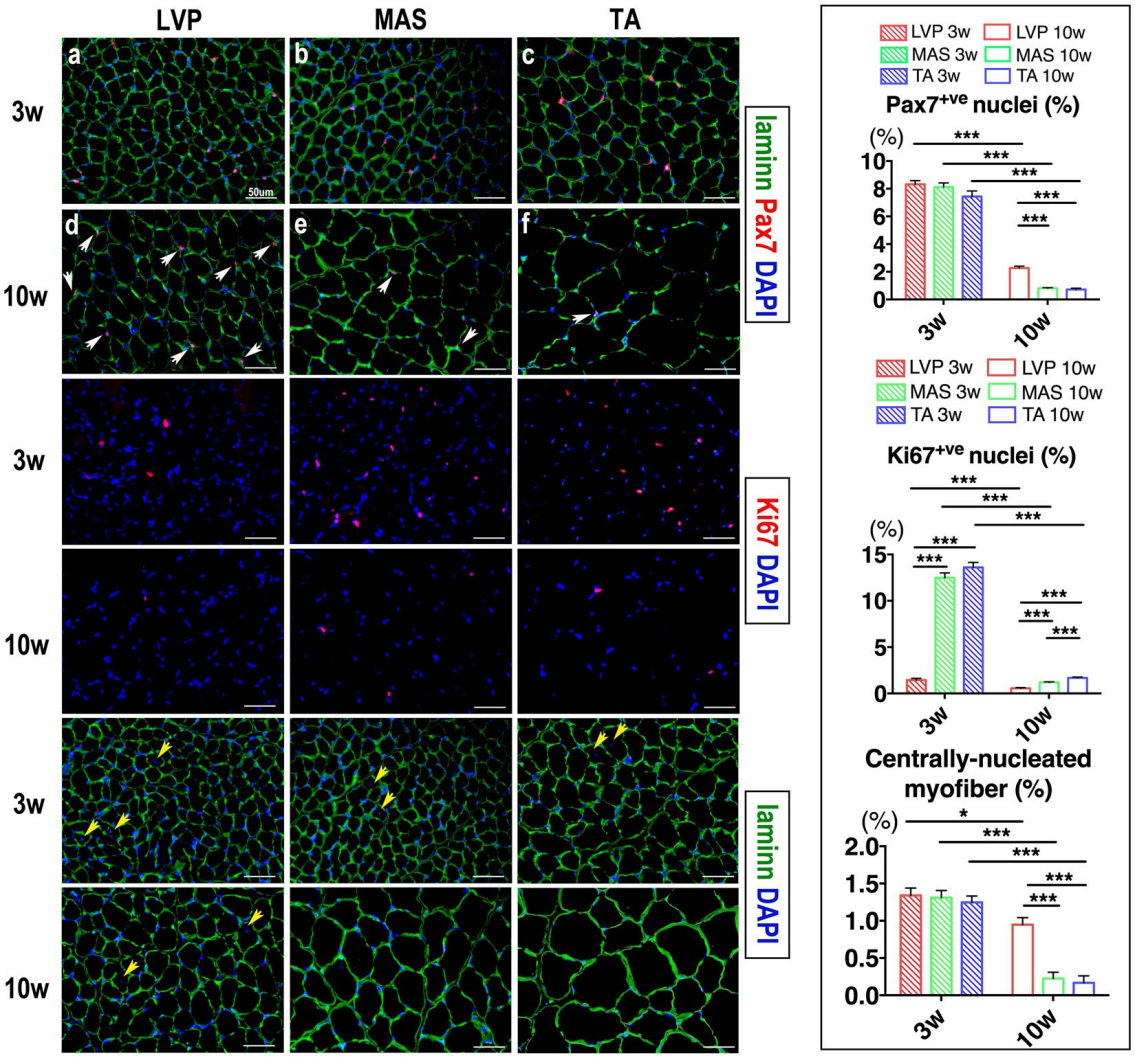
Regeneration-related activity in LVP, MAS and TA muscle. (**a**–**f**) Immunofluorescence staining of laminin (green), Pax7 (red) and DAPI (blue) in muscle cryosections from LVP, MAS and TA muscle, at either three-week age or ten-week age. White arrows indicate Pax7^+ve^ nuclei. (**g**,**h**) Quantification of the percentage of Pax7^+ve^ nuclei in total nuclei. (**i**–**n**) Immunofluorescence staining of Ki67 (red) and DAPI (blue) in muscle cryosections from LVP, MAS and TA, at either three-week age or ten-week age. (**o**,**p**) Quantification of the percentage of Ki67^+ve^ nuclei in total nuclei. (**q**–**v**) Immunofluorescence staining of laminin (green) and DAPI (blue) in muscle cryosections from LVP, MAS and TA, at either three-week age or ten-week age. Yellow arrows indicate centrally-nucleated myofibers. (**w**,**x**) Quantification of the percentage of centrally-nucleated myofiber in total myofibers. For each group, N = 6. *p < 0.05; ***p < 0.001.

Satellite cells (SCs) are resident muscle stem cells and play a pivotal role in the initiation of muscle growth and regeneration^19^. Pax7, the specific marker for SCs, was used to map SCs population within the three muscles. The percentage of Pax7^+ve^ nuclei was similar among the three muscles at three weeks, but was significantly higher in LVP muscle than in MAS and TA muscle (*p* = 0.000, *p* = 0.000, Fig. 1a–f, quantified in g) at ten weeks. The percentage of Ki67^+ve^ nuclei was highest in TA muscle and lowest in LVP muscle with substantial differences between each group, both at three weeks and ten weeks (Fig. 1i–n, quantified in o). Similar to SCs, the percentage of centrally-nucleated myofibers (CNMs) was comparable among the three muscles at three weeks, but was about five times higher in LVP muscle than in MAS and TA (Fig. 1q–v, quantified in w). During post-natal growth, the percentage of both SCs and CNMs decreased considerably among all three muscles (Fig. 1h,x). The mitotic activity substantially decreased with age in LVP, MAS and TA muscle, but the decline was 67% in LVP muscle, yet nearly 90% in MAS and TA muscle (Fig. 1p).

### Wnt7a expands muscle satellite cell population *in vivo*

**Figure 2:**
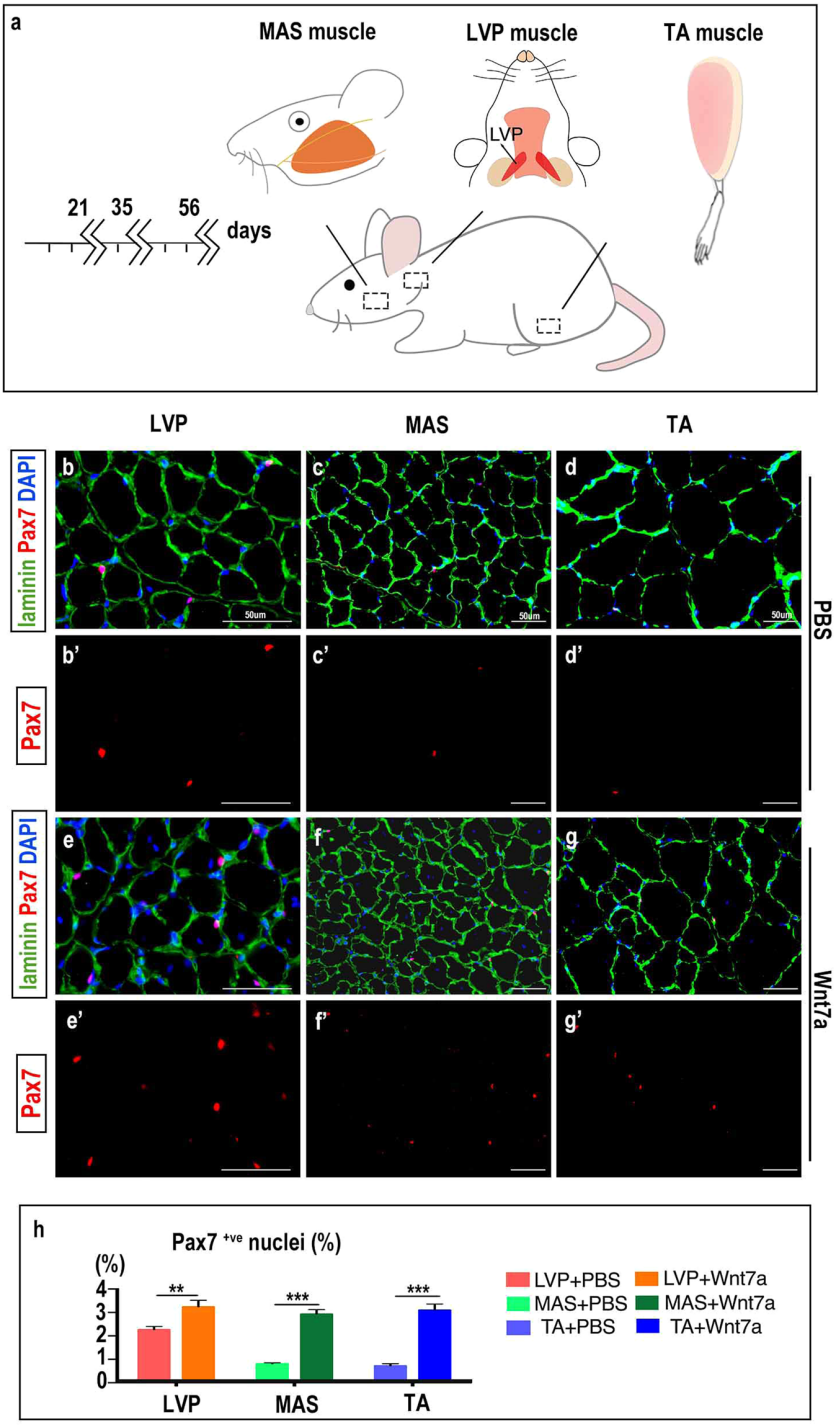
Wnt7a increases satellite cell proportion. (**a**) Schematic illustration of experimental procedures: Rat LVP, MAS, TA muscle were injected with rh-Wnt7a and were analyzed at day 21, 35, 56 after rh-Wnt7a administration. (**b**–**d**) Immunofluorescence staining of laminin (green), Pax7 (red) and DAPI (blue) in PBS-treated muscle groups. (b’–d’) Immunofluorescence staining Pax7 (red) in PBS-treated muscle groups. (**e**–**g**) Immunofluorescence staining of laminin (green), Pax7 (red) and DAPI (blue) in rh-Wnt7a-treated muscle groups. (e’–g’) Immunofluorescence staining Pax7 (red) in Wnt7a-treated muscle groups. (**h**) Quantification of Pax7^+ve^ nuclei in PBS-treated and rh-Wnt7a-treated muscle groups at 21 days after injection. For each group, N = 6. **p < 0.01; ***p < 0.001.

A single doze of rh-Wnt7a was injected into LVP, MAS, or TA muscle of ten-week adult rats, and samples were examined at three, five and eight weeks after injection (Fig. 2a). At three weeks after injection, the proportion of SCs in rh-Wnt7a-treated groups was significantly higher than in the PBS controls (1.43 fold in LVP muscle, *p* = 0.003; 3.62 fold in MAS muscle, *p* = 0.000; 4.31 fold in TA muscle, *p* = 0.000, Fig. 2b–g,b’–g’, quantified in h). At later time points, the percentage of Pax7^+ve^ went back to basal level in LVP muscle and TA, but remained two-time higher in MAS (Supplemental Fig. S1). Considering the confirmed correlation between the size of SCs population and fibre type composition^2^, we set out to explore the potential switch in fibre types after rh-Wnt7a delivery.

### Wnt7a induces different modes of fibre type switch among muscles

**Figure 3:**
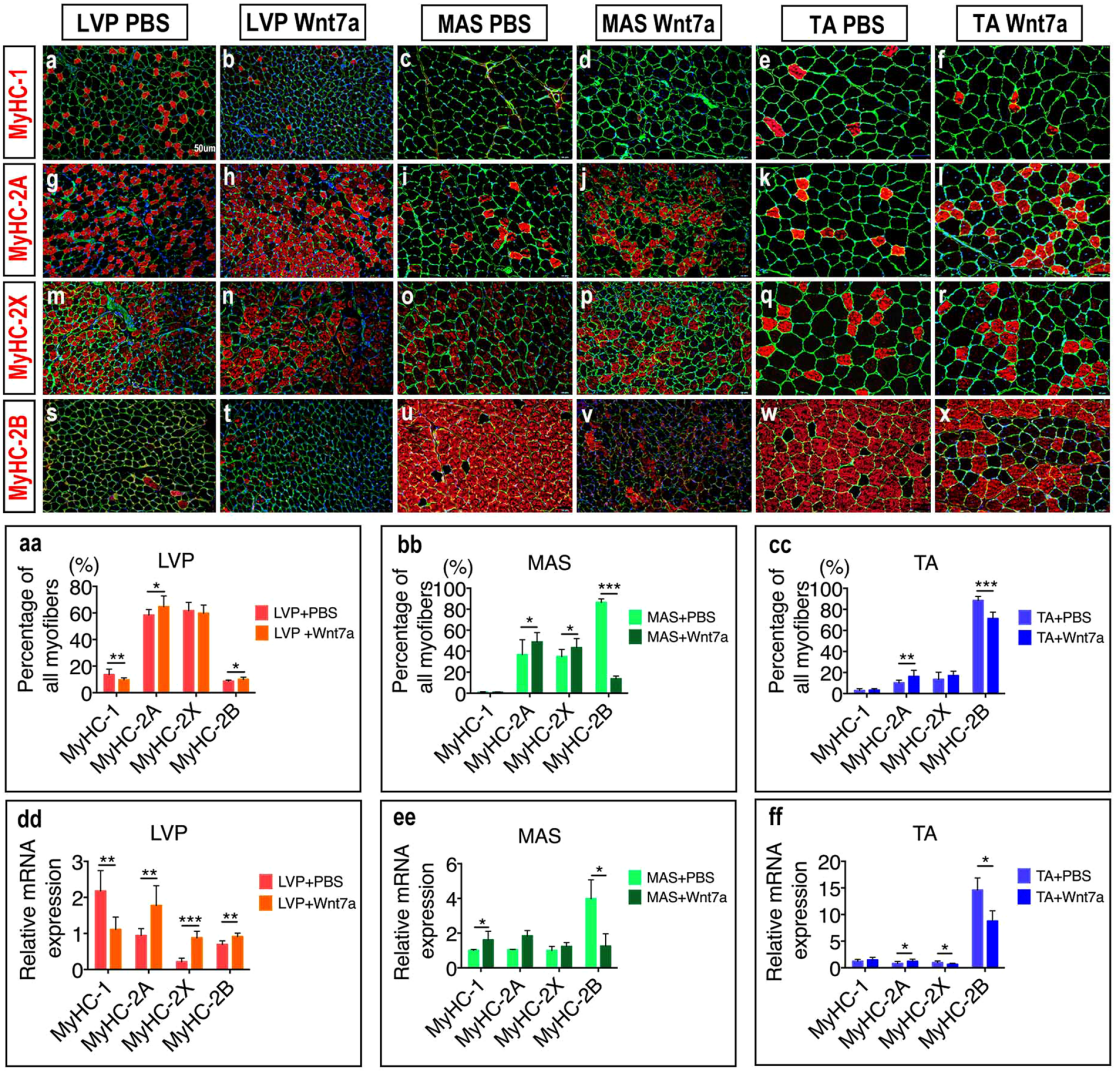
Wnt7a induces different fiber type switch patterns in LVP muscle, MAS and TA muscles. (**a**–**f**) Immunofluorescence staining of laminin (green), MyHC-1 (red) and DAPI (blue) in PBS-treated and rh-Wnt7a-treated muscle groups. (**g**–**l**) Immunofluorescence staining of laminin (green), MyHC-2A (red) and DAPI (blue) in PBS-treated and rh-Wnt7a-treated muscle groups. (**m**–**r**) Immunofluorescence staining of laminin (green), MyHC-2X (red) and DAPI (blue) in PBS-treated and rh-Wnt7a-treated muscle groups. (**s**–**x**) Immunofluorescence staining of laminin (green), MyHC-2B (red) and DAPI (blue) in PBS-treated and rh-Wnt7a-treated muscle groups. (aa,bb,cc) Quantification of the proportion of MyHC-1^+ve^, MyHC-2A^+ve^, MyHC-2X^+ve^, MyHC-2B^+ve^ myofibers in LVP MUSCLE muscle, MAS muscle and TA muscle. (dd,ee,ff) Quantification of relative mRNA expression of MyHC-1, MyHC-2A, MyHC-2X, MyHC-2B in LVP muscle, MAS muscle and TA muscle. For each group, N = 6. *p < 0.05; **p < 0.01; ***p < 0.001.

Depending on their metabolism and MyHC expression, skeletal muscle fibres are divided into four main types: MyHC-1, MyHC-2A, MyHC-2X and MyHC-2B. MyHC-1 is classified as slow fibres, whereas the other three as fast fibers^20^. At three weeks after rh-Wnt7a delivery, a significant decrease in the proportion of MyHC-1^+ve^ slow fibres was observed in LVP muscle (*p* = 0.005), but not in MAS muscle (*p* = 0.757) or TA muscle (*p* = 0.543) (Fig. 3a–f,aa–cc). The proportion of MyHC-2A^+ve^ fast fibres significantly increased in LVP muscle (*p* = 0.024), MAS muscle (*p* = 0.021) and TA muscle (*p* = 0.004) (Fig. 3g–l,aa–cc). On the other hand, the proportion of MyHC-2B^+ve^ fast fibres significantly increased in LVP muscle (*p* = 0.019), but decreased significantly in both MAS (*p* = 0.000) and TA (*p* = 0.000) (Fig. 3s–x,aa–cc). Generally, there existed a slow-fast switch in the myofiber composition in LVP muscle after rh-Wnt7a stimulus, but a fast-slow switch in MAS and TA muscle. These fibre type switches were confirmed by mRNA expression analysis (Fig. 3dd,dd,ff).

### Wnt7a promotes both hyperplasia and hypertrophy in myofibers

**Figure 4:**
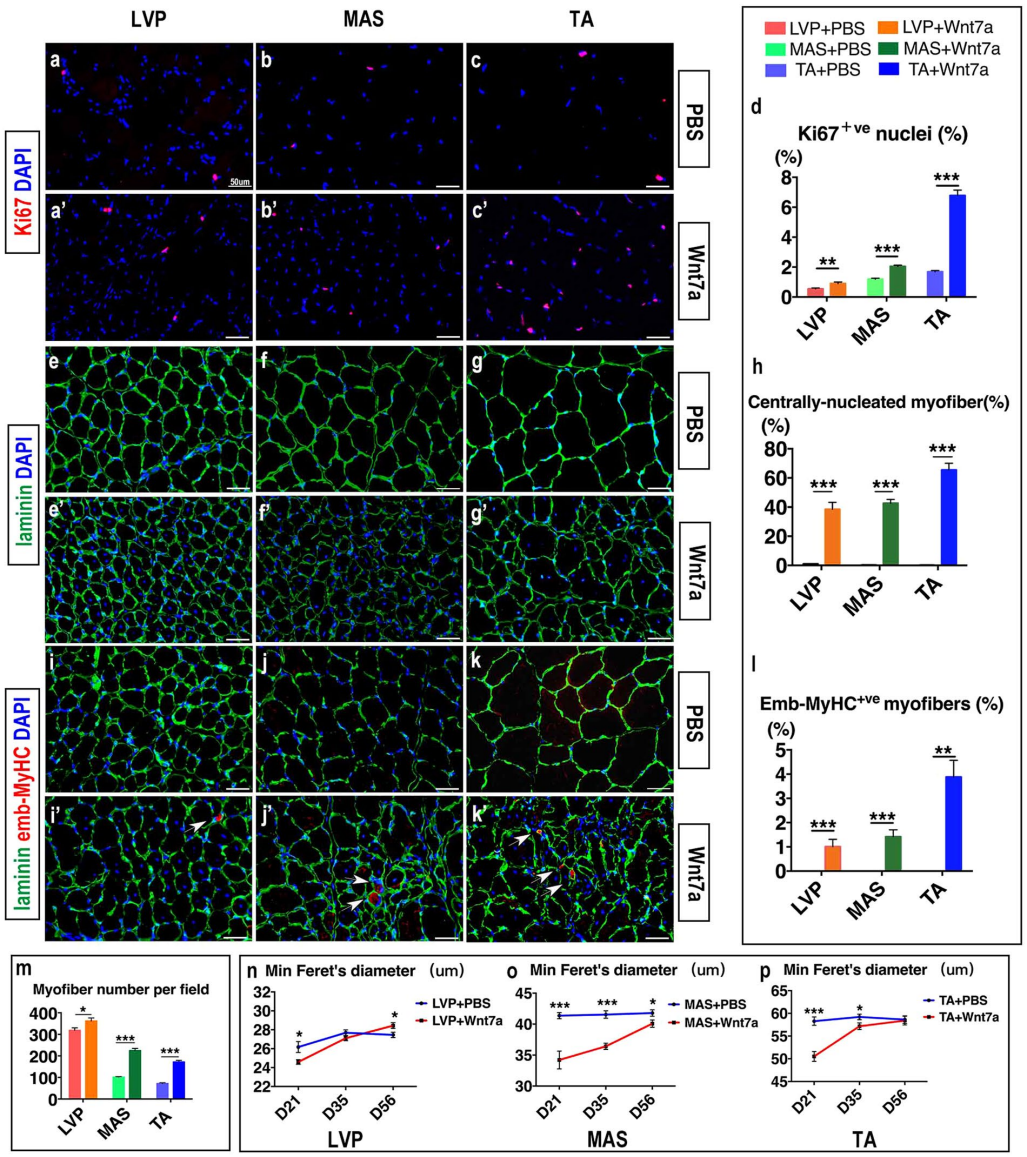
Wnt7a promotes myofiber hyperplasia and hypertrophy. (**a–c**,a’–c’) Immunofluorescence staining of Ki67 (red) and DAPI (blue) in PBS-treated and rh-Wnt7a-treated muscle groups, respectively. (**d**) Quantification of Ki67^+ve^ nuclei in PBS-treated and rh-Wnt7a-treated muscle groups. (**e**–**g**,e’–g’) Immunofluorescence staining of laminin (green) and DAPI (blue) in PBS-treated and rh-Wnt7a-treated muscle groups, respectively. (**h**) Quantification of centrally-nucleated myofibers in PBS-treated and rh-Wnt7a-treated muscle groups. (**i**–**k**,i’–k’) Immunofluorescence staining of emb-MyHC (red), laminin (green) and DAPI (blue) in PBS-treated and rh-Wnt7a-treated muscle groups, respectively. White arrows indicate emb-MyHC^+ve^ myofibers. (**l**) Quantification of emb-MyHC^+ve^ myofibers in PBS-treated and rh-Wnt7a-treated muscle groups. (**m**) Quantification of total myofibers per microscopic field in PBS-treated and rh-Wnt7a-treated muscle groups. (**n**) Quantification of Min Ferret’s Diameter in PBS-treated and rh-Wnt7a-treated LVP muscle at day 21, 35, 56. (**o**) Quantification of Min Ferret’s Diameter in PBS-treated and rh-Wnt7a-treated MAS muscle at day 21, 35, 56. (**p**) Quantification of Min Ferret’s Diameter in PBS-treated and rh-Wnt7a-treated TA muscle at day 21, 35, 56. For each group, N = 6. *p < 0.05; **p < 0.01; ***p < 0.001.

At three weeks after rh-Wnt7a injection, the percentage of Ki67^+ve^ proliferating cells was significantly higher in all three muscles than their PBS controls (1.63 fold in LVP muscle, *p* = 0.003; 1.70 fold in MAS muscle, *p* = 0.000; 4.02 fold in TA muscle, *p* = 0.000, Fig. 4a–c,a’–c’, quantified in d). CNMs were almost absent in PBS controls, but their percentage among total myofibers reached 38.52 ± 4.73% in LVP muscle, 42.63 ± 2.64% in MAS muscle and 65.50 ± 4.59% in TA muscle (Fig. 4e–g,e’–g’, quantified in h) with rh-Wnt7a injection. At the same time, among all three muscles, rh-Wnt7a injection induced the emergence of myofibers expressing embryonic isoform of myosin heavy chain (emb-MyHC), the percentage of which among total myofibers was 1.01 ± 0.30% in LVP muscle, 1.42 ± 0.28% in MAS muscle, and 3.88 ± 0.69% in TA muscle (Fig. 4i–k,i’–k’, quantified in l). In accordance, among all three muscles, the myofiber density in Wnt7a groups was significantly higher when compared with PBS controls (*p* = 0.023 in LVP, *p* = 0.000 in MAS, *p* = 0.000 in TA, Fig. 4m). Overall, rh-Wnt7a injection induced substantial hyperplasia in all three muscles. When observed at later time points, the percentage of both Ki67^+ve^ cells and CNMs, as well as the density of myofibers, started to decrease in all three muscles (Supplemental Fig. S1).

Meanwhile, the average min Ferret’s diameter of myofibers at three weeks was significantly smaller in Wnt7a groups than controls (*p* = 0.015 in LVP, *p* = 0.000 in MAS, *p* = 0.000 in TA, Fig. 4e–g,e’–g’, quantified in n-p), but increased steadily in the following weeks among all three muscles (Fig. 4n–p). At eight weeks after rh-Wnt7a injection, when compared with PBS control, the min Ferret’s diameter was even greater in LVP muscle (Fig. 4n), but smaller in MAS muscle (Fig. 4o) and comparable in TA muscle (Fig. 4p). These data suggested evident myofiber hypertrophy following initial hyperplasia with rh-Wnt7a stimulus.

### Wnt7a induces location-specific alterations in regeneration-related pathways

According to previous theories established on data acquired from limb muscles, Wnt7a induced SCs expansion via Vangl2 mediated Planar Cell Polarity (PCP) pathway^15^, and hypertrophy via Akt/mTOR pathway^14^. In addition, myofiber hyperplasia has been associated with down-regulation of myostatin pathway^21^.

**Figure 5:**
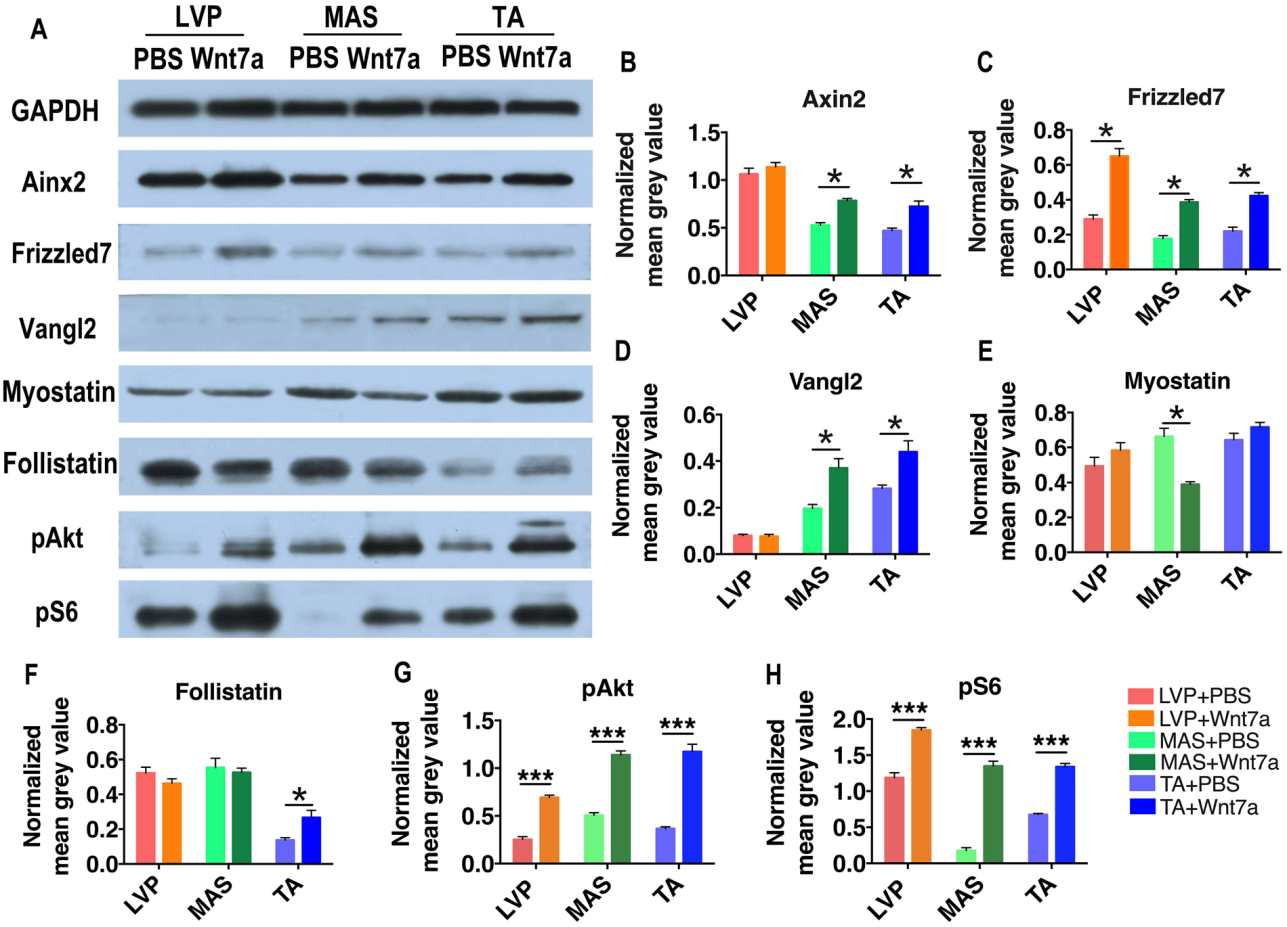
Wnt7a induces location-specific changes in regeneration-related pathways. (**A**) Western blot analysis of core proteins in muscle regeneration-related pathways. Representative blots were shown in PBS-treated and rh-Wnt7a-treated muscle groups. (**B**–**H**) Densitometric analysis of immunoblots of Axin2, Frizzled7, Vangl2, Myostatin, Follistatin, pAkt and pS6. Full-length gels and blots are included in Supplemental Fig. S3. For each group, N = 6. *p < 0.05, ***p < 0.001.

At protein level, rh-Wnt7a injection led to significantly higher expression of Axin2 in MAS muscle and TA muscle but not in LVP muscle (*p* = 0.028 in MAS, *p* = 0.017 in TA, *p* = 0.393 in LVP, Fig. 5A,B), significantly higher expression of Frizzled7 in all three muscles (*p* = 0.017 in LVP, *p* = 0.019 in MAS, *p* = 0.022 in TA, Fig. 5A,C), and significantly higher expression of Vangl2 in MAS and TA but not in LVP muscle (*p* = 0.017 in MAS, *p* = 0.034 in TA, *p* = 0.767 in LVP, Fig. 5A,D). For myostatin pathway, rh-Wnt7a injection led to significantly lower expression of myostatin in MAS (*p* = 0.032, Fig. 5A,E) and significantly higher expression of follistatin in TA (*p* = 0.042, Fig. 5A,F) when compared with PBS controls. No significant difference was detected in other muscle groups. The expression of pAkt and pS6, critical components of Akt/mTOR pathway, was substantially higher in rh-Wnt7a-treated LVP muscle, MAS and TA when compared with their corresponding controls (Fig. 5A,G,H).

## Discussion

In view of embryonic origin, skeletal muscle has been broadly divided into two categories: branchiomeric muscle and somite-derived muscle. Somite-derived muscle includes all the limb and trunk muscles, while branchiomeric muscle comprises the majority of head muscles^22^. Multiple studies have illuminated significant differences between somite-derived muscle and branchiomeric muscle in their regenerative or reparative responses^11,18,23^. Our previous study in rat demonstrated that LVP muscle was of distinct histological features and myofiber composition, compared with TA muscle, and exogenous Wnt7a induced different levels of hyperplasia in LVP muscle and TA muscle^16^. In the present study, we further studied the differences in the responses and mechanisms underlying the alterations after rh-Wnt7a administration in LVP muscle, MAS muscle and TA muscle.

SCs are muscle specific stem cells wedged between basal lamina and sarcolemma. Although accounting for less than 1% of total nuclei in uninjured muscle^24^, they are believed to be indispensable in skeletal muscle growth and regeneration. SCs are generally believed to be quiescent under basal conditions. When activated, satellite cells proliferated and fused into existing myofibers, producing CNMs or forming *de novo* myofibers^25^. Our study revealed that, early after birth, all three muscles are of comparable density of SCs and CNMs, which dropped during the postnatal development process. At ten-week age, SCs proportion in LVP muscle were much more than 1%, yet remaining well below 1% in MAS muscle and TA muscle. Heterogeneities in SCs proportion among muscles of different embryonic origins or different ages were also reported by other studies^11,26,27^. It has been proved by Carvajal Monroy that rat LVP muscle and MAS muscle contained more SCs than limb muscle^27^. Keefe reported that the SCs density varied nearly five folds among different muscles^26^. Randolph demonstrated that SCs in pharyngeal muscles were of a larger population and undergo more active myonuclear turnover than in limb muscles^11^. For all three muscles, rh-Wnt7a stimulus was efficient in expanding the SCs population and subsequently increasing the density of CNMs, suggesting its therapeutic potential in the craniofacial region. The vigorous SC increase after rh-Wnt7a delivery was believed to be a result of activated PCP signalling, which mediated up-regulation and polarized localization of Vangl2 and thus promoted SC symmetric expansion^15^.

Fibre type composition varies according to the functional demands of each specific muscle. MAS muscle and TA muscle are generally considered as fast muscles and mainly composed of MyHC-2B fibres, since they undertake movements requiring strong contractions^2^. Carvajal Monroy reported that the LVP muscle was exclusively composed of fast fibers^28^, but our work revealed 13.6% slow fibres existing in LVP muscle, which was in accordance with Furusawa’s work^29^. The differences might be attributed to different muscle harvest procedures. Carvajal Monroy took the entire posterior part of soft palate, which also included muscles other than LVP muscle in the soft palate region. In contrast, the dissection method in our work and Furusawa’s managed sole isolation of LVP muscle for analyses.

Interestingly, rh-Wnt7a induced different myofiber switch patterns among the three muscles. Fast-to-slow fibre switch appeared in TA and MAS muscle, which was in accordance with von Maltzahn’s data^30^, but a slow-to-fast fibre switch occurred in LVP muscle. Different fibre type composition and unique properties^2^ in LVP, MAS and TA muscle might underlie the antithetical switch direction. We noted that although the SCs density reached comparable level among the three muscles, the increase was significantly less robust in LVP muscle. Such difference might be associated with the specific myofiber type switch pattern in it. Since a higher percentage of SCs has been found in slow fibres than in fast fibers^20^, the slow-fast fibre shift in LVP muscle might be accountable for the less robust SCs increase. On the other hand, different fibre types have their unique clinical significance. For example, inducing slow muscle fibre could ameliorate Duchenne muscular dystrophy^30^; while enhancing the growth of fast fibre could act against aging-related muscle loss^20^. Taking account of skeletal muscle fibre type plasticity, fibre types could be remodelled to meet different clinical needs.

Remarkably, our experiment demonstrated that rh-Wnt7a vigorously promoted myofiber hyperplasia in skeletal muscle, as evidenced by an elevated level of Ki67^+ve^ nuclei, emb-MyHC expression and myofiber density. It is the first time that Wnt7a had been proved effective in inducing myofiber hyperplasia. In rats, postnatal muscle growth after ten weeks was mainly due to an increase in myofiber size while fibre number remained constant^31^. Consequently, the increase of myofiber number observed here was assumed to be the direct effect of rh-Wnt7a administration. Emb-MyHC was reported to express in newly-formed regenerating myofibers rather than mature adult myofibers^2^, indicating that rh-Wnt7a induced production of new myofibers. Typically, in muscle regeneration process, where muscle injury was initiated, embryonic isoform of myosin heavy chain began to express in newly-formed regenerating myofibers at 2-3 days after injury and the expression became barely noticeable at 21-day post injury. In the experiment conducted here, no injury was introduced prior to Wnt7a delivery and the expression of emb-MyHC was detected at day21, yet at a low level^32^. Since it was reported in Le Grand’s work that rh-Wnt7a had no effect on myogenic proliferation^15^, one possible explanation was that rh-Wnt7a might exert an effect on interstitial cells, say, PW1^+^ cells, which was demonstrated to experience a large increase during regeneration^33^.

The increase in myofiber density was concomitant with an initial decrease in myofiber diameter. When observed at later time points, the fibres gradually enlarged, leading to significant hypertrophy in LVP muscle observed at D56. The fibre diameter in TA and MAS muscle was, however, not larger than control group. LVP muscle is, at least, more amenable to rh-Wnt7a-mediated fibre growth. Furthermore, it was revealed the expression level of pAkt and pS6 in all the three muscles was significantly elevated. The corollary is that Wnt7a/Akt/mTOR pathway was activated and the increase in fibre diameter had not yet demonstrated. In addition, the proportion of CNMs and Ki67^+ve^ nuclei remained considerably higher than control group even at D56, when muscle regeneration process was believed to fully complete^34^. The prospect for further growth in TA and MAS muscle after D56 was thus possible.

Myofiber hyperplasia could be seen as a beneficial adaptation and myostatin has been strongly implicated in the hyperplasia of skeletal muscle fibers^21^. Myostatin, also called growth differentiation factor-8 (GDF-8), is able to inhibit myoblast differentiation and down-regulate skeletal muscle size^35^. Our results revealed that the hyperplasia group did experience a significant decrease in myostatin expression, further confirming this negative correlation. In addition, a significant increase in follistatin, an efficacious antagonist of myostatin, was detected in the hyperplasia group. Similarly, Medeiros and his colleagues had reported that overexpression of follistatin could cause myofiber hyperplasia^36^. The significant alterations in myostatin pathway after rh-Wnt7a administration lend credence to the interaction between myostatin pathway and Wnt pathway, which is labyrinthine and just beginning to be characterized. Myostatin pathway and Wnt pathway are among the most important pathways regulating skeletal muscle fibre growth. Myostatin, characterized as the muscle chalone^37^, was believed to be negatively associated with myofiber growth. Wnt signalling mediated myofiber growth via canonical and non-canonical pathways^38^. Traditionally, it is believed that Follistatin induced myofiber hypertrophy by antagonizing myostatin-mediated repressive effects on muscle fibre growth. Recent work suggested that follistatin possessed a myostatin-independent function and could be controlled by Wnt signaling^39^. Briefly, in addition to the two verified Wnt7a-mediated non-canonical pathways in promoting myogenesis, we propose here a third one: Wnt7a could induce myofiber hyperplasia, by interacting with myostatin pathway (Supplemental Fig. S2).

There are several limitations in this study. First, the results were obtained from single doze injection of rh-Wnt7a and the injection doze was somehow arbitrarily determined based on the muscle size. Second, branchiomeric head muscles not only distinguished from somite-derived limb muscles, but also exhibited heterogeneity among head muscles themselves. Data acquired from head muscles other than LVP muscle and MAS is imperative in future studies to fully depict the features of branchiomeric muscle population. Also, since muscle regeneration process typically involves myofiber necrosis, which was not included in our study, the elevated cell proliferation activity we observed could be more safely called muscle regeneration-related phenotype rather than muscle regeneration. In order to characterize the specific regenerative effect rh-Wnt7a would exert on these muscles, an injury model should be employed.

In conclusion, both craniofacial and limb muscles could be effectively expanded by exogenous Wnt7a stimulus, but muscle-to-muscle variations in response patterns existed. This study offers a fresh prospective in skeletal muscle regeneration research and provides a biological foundation for bio-therapeutics in craniofacial muscle regeneration.

## Materials and Methods

### Animals

Adult male Sprague-Dawley rats (10 weeks, 280–300 g) were were purchased from Dashuo Biological Technology Company, Chengdu, China. All rats were raised in a humidity-controlled (53 ± 2%) and temperature-controlled (23 ± 2 °C) facility and were on a 12-h light/dark cycle. A total of 60 animals were used in this study and animal distribution was listed in Supplemental Table S1. All experimental procedures on animals were in accordance with National Institute of Health Guidelines for the Care and Use for Laboratory animals and were approved by the Institutional Animal Care and Use Committee (IACUC, protocol number: WCCSIB-D-2014-007) at Sichuan University.

### Intramuscular rh-Wnt7a delivery

In this study, LVP muscle from the velopharynx and MAS muscle were representatives for craniofacial muscle, and TA muscle was chosen as a representative for limb muscle. Rats were anesthetized with intramuscular injection of Zoletil (50 mg/kg) plus atropine (0.05 mg/kg). Rh-Wnt7a (R&D systems) was injected directly into the muscles. For each TA (n = 6), 75 μl rh-Wnt7a (100 μg/ml) was delivered; for each MAS (n = 6), 75 μl rh-Wnt7a (100 μg/ml) was delivered; for each LVP muscle (n = 6), 25 μl rh-Wnt7a (100 μg/ml) was injected. The contralateral muscle of the same animal was injected with an equal volume of phosphate-buffered saline (PBS) as control. Rats were euthanized with diethyl ether inhalation, followed by decapitation.

### Muscle dissection

TA muscle was cut from tendon to tendon on the tibia anterior bone. Superficial MAS muscle was dissected from its origin in the anterior portion of the cranium to its insertion on the posterior portion of the mandible^40^. LVP muscle was dissected and isolated according to Carvajal Monroy^28^ with some modifications. A ventral skin incision extending from the mandibular symphysis to the clavicle was made. After careful dissection of the posterior digastric muscle, sternocleidomastoid muscle and stylohyoid muscle, the levator veli palatini muscle became visible with its tendon closely attached to tympanic bulla. LVP muscle was carefully isolated and harvested. Muscle samples were harvested at 21, 35, and 56 days after rh-Wnt7a injection.

### Immunofluorescence

Muscle Cryosections were made using Meng’s method^41^. Sections were incubated with blocking serum, and were subsequently incubated at 4 °C overnight with primary antibodies. Then the sections were incubated for 1 h at room temperature with corresponding secondary antibodies at room temperature for one hour. Images were captured with Olympus BX63 immunofluorescence microscope. Muscle cryosections were made at 10 μm thickness and fixed in 0 °C acetone (100%) for 20 min. Sections were blocked with phosphate-buffered saline (PBS) containing 5% bovine serum albumin and 5% donkey serum for 1 h at 25 °C, and subsequently incubated overnight at 4 °C with primary antibodies. Following washing in PBS, sections were incubated for 1 h at 25 °C with Alexa Fluor 488-conjugated and 568-conjugated secondary antibodies (Invitrogen; Thermo Fisher Scientific, Inc., Waltham, MA, USA). After several washes in PBS, the nuclei were stained with 4′,6-diamidino-2-phenylindole (DAPI). Images were captured with an Olympus BX63 fluorescence microscope (Olympus Corporation, Tokyo, Japan). The primary antibodies used in this study were listed in Supplemental Table S2.

### Quantitative RT-PCR

Muscle sample homogenization and RNA extraction was conducted using Trizol reagents (Invitrogen, USA). First-strand cDNA was synthesized using Rayscript cDNA Synthesis KIT (GENEray, GK8030, Shanghai, China). Real-time PCR was performed using AceQ^TM^ qPCR Probe Master Mix (Q112-02, Bio-Rad, USA) in an ABI 7500 system. The comparative cycle threshold (CT) was used to analyse the data by generating relative values of the amount of target cDNA as described^42^. The expression level was normalized to ACTB. Primers used are listed in Supplemental Table S3. All experiments were done in triplicate.

### Western Blot

Muscle samples were minced and quickly prepared in RIPA lysis buffer. Tissues were incubated for 30 min on ice, followed by centrifugation at 12,000 × g for 10 min at 4 °C. Total protein concentration in the supernatant was determined using a BCA protein assay kit (Beijing Solarbio Science & Technology Co., Ltd.). Protein extract (7 μl/lane) was loaded on 10% SDS-PAGE and transferred onto polyvinylidene fluoride membranes. After blocking with 5% bovine serum albumin in 0.5% TBS-Tween-20 at room temperature for 1 h, the membranes were incubated with primary antibodies at 4 °C overnight. Then, the membranes were incubated with horseradish peroxidase-conjugated secondary antibody for 1 h at 37 °C. The protein bands were visualized using an enhanced chemiluminescence system. Densitometry values were normalized to the intensity of corresponding bands for GAPDH. Quantitative analysis of western blotting was performed using Adobe Photoshop and ImageJ. The primary antibodies used in this study were listed in Supplemental Table S2.

### Quantification and statistical analyses

Quantification of cell numbers and myofiber diameter were performed using ImageJ. All data were analysed using SPSS 19.0 or Graphpad Prism. Data distribution was tested with one-sample Kolmogorov-Smirnov test. Results were presented as mean ± SEM. A two-tailed independent Student’s t-test was used to evaluate statistical differences between PBS-treated and rh-Wnt7a-treated muscles (n = 12) in each muscle group. One-way ANOVA with Turkey *post hoc* analysis was used to analyse the differences among the three muscle groups (n = 12). *p* < 0.05 was considered statistically significant.

## Electronic supplementary material

Supplemental files

## References

[1] Randolph ME, Pavlath GK (2015). A muscle stem cell for every muscle: variability of satellite cell biology among different muscle groups. Front Aging Neurosci.

[2] Schiaffino S, Reggiani C (2011). Fiber types in mammalian skeletal muscles. Physiol Rev.

[3] Tabebordbar M, Wang ET, Wagers AJ (2013). Skeletal muscle degenerative diseases and strategies for therapeutic muscle repair. Annu Rev Pathol.

[4] Kotlarek KJ, Perry PL, Fang X (2017). Morphology of the Levator Veli Palatini Muscle in Adults With Repaired Cleft Palate. J. Craniofac. Surg..

[5] Ha S, Kuehn DP, Cohen M, Alperin N (2007). Magnetic resonance imaging of the levator veli palatini muscle in speakers with repaired cleft palate. Cleft Palate Craniofac J.

[6] Tian W (2010). Magnetic Resonance Imaging Assessment of Velopharyngeal Structures in Chinese Children After Primary Palatal Repair. J Craniofac Surg.

[7] Lu Y, Shi B, Zheng Q, Xiao W, Li S (2006). Analysis of velopharyngeal morphology in adults with velopharyngeal incompetence after surgery of a cleft palate. Ann Plast Surg.

[8] Perry JL, Kuehn DP, Sutton BP, Goldwasser MS, Jerez AD (2011). Craniometric and velopharyngeal assessment of infants with and without cleft palate. J. Craniofac. Surg..

[9] Kane AA, Lo L, Christensen GE, Vannier MW, Marsh JL (1996). Relationiship between Bone and Muscles of Mastication in Hemifacial Microsomia. Plast Reconstr Surg.

[10] Sambasivan R, Kuratani S, Tajbakhsh S (2011). An eye on the head: the development and evolution of craniofacial muscles. Development.

[11] Randolph ME (2015). Pharyngeal Satellite Cells Undergo Myogenesis Under Basal Conditions and Are Required for Pharyngeal Muscle Maintenance. Stem Cells.

[12] Chakravarthy MV, Davis BS, Booth FW (2000). IGF-1 restores satellite cell proliferative potential in immobilized old skeletal muscle. J Appl Physiol.

[13] Borselli C (2010). Functional muscle regeneration with combined delivery of angiogenesis and myogenesis factors. Proc Natl Acad Sci USA.

[14] von Maltzahn J, Bentzinger CF, Rudnicki M (2012). Wnt7a-Fzd7 signalling directly activates the Akt/mTOR anabolic growth pathway in skeletal muscle. Nat Cell Biol.

[15] Le Grand F, Jones AE, Seale V, Scime A, Rudnicki M (2009). Wnt7a activates the planar cell polarity pathway to drive the symmetric expansion of satellite stem cells. Cell Stem Cell.

[16] Cheng X, Song L, Lan M, Shi B, Li J (2017). Morphological and molecular comparisons between tibialis anterior muscle and levator veli palatini muscle: A preliminary study on their augmentation potential. Exp. Ther. Med..

[17] Cohen SR, Chen L, Trotman C, Burdi AR (1993). Soft-palate myogenesis: a devolopmental field paradigm. Cleft Palate Craniofac J.

[18] Ono Y, Boldrin L, Knopp P, Morgan JE, Zammit P (2010). Muscle satellite cells are a functionally heterogeneous population in both somite-derived and branchiomeric muscles. Dev Biol.

[19] Chang NC, Rudnicki M (2014). Satellite cells: the architects of skeletal muscle. Curr Top Dev Biol.

[20] Talbot J, Maves L (2016). Skeletal muscle fiber type: using insights from muscle developmental biology to dissect targets for susceptibility and resistance to muscle disease. Wiley Interdiscip Rev Dev Biol.

[21] Johnston APW, Bellamy LM, de Lisio M, Parise G (2011). Captopril treatment induces hyperplasia but inhibits myonuclear accretion following severe myotrauma in murine skeletal muscle. Am. J. Physiol. Regul. Integr. Comp. Physiol..

[22] Diogo R (2015). A new heart for a new head in vertebrate cardiopharyngeal evolution. Nature.

[23] Pavlath GK (1998). Heterogeneity among muscle precursor cells in adult skeletal muscles with differing regenerative capacities. Dev Dyn.

[24] Pawlikowski B, Vogler TO, Gadek K, Olwin BB (2017). Regulation of skeletal muscle stem cells by fibroblast growth factors. Developmental Dynamics.

[25] Yin H, Price F, Rudnicki M (2013). Satellite cells and the muscle stem cell niche. Physiol Rev.

[26] Keefe AC (2015). Muscle stem cells contribute to myofibres in sedentary adult mice. Nat Commun.

[27] Carvajal Monroy PL, Grefte S, Kuijpers-Jagtman AM, Von den Hoff JW, Wagener FADT (2017). Neonatal Satellite Cells Form Small Myotubes *In Vitro*. J. Dent. Res..

[28] Carvajal Monroy PL (2013). A rat model for muscle regeneration in the soft palate. PLoS One.

[29] Yasuda K (2003). Central distribution of neuronal cell bodies innervating the levator veli palatini muscle and associated pattern of myosin heavy chain isoform expression in rat. Brain Res..

[30] von Maltzahn J, Renaud J, Parise G, Rudnicki M (2012). Wnt7a treatment ameliorates muscular dystrophy. Proc. Natl. Acad. Sci..

[31] Tamaki T, Akatsuka A, Yoshimura S, Roy RR, Edgerton AR (2002). New fiber formation in the interstitial space of rat skeletal muscle during postnatal growth. J. Histochem. Cytochem..

[32] Schiaffino S, Rossi A, Smerdu V, Leinwand LA, Reggiani C (2015). Developmental myosins: expression patterns and functional significance. Skelet Muscle.

[33] Mitchell KJ (2010). Identification and characterization of a non-satellite cell muscle resident progenitor during postnatal development. Nat. Cell Biol..

[34] Hardy D (2016). Comparative Study of Injury Models for Studying Muscle Regeneration in Mice. PLoS One.

[35] Snijders T (2015). Satellite cells in human skeletal muscle plasticity. Front Physiol.

[36] Medeiros EF, Phelps MP, Fuentes FD, Bradley TM (2009). Overexpression of follistatin in trout stimulates increased muscling. Am. J. Physiol. Regul. Integr. Comp. Physiol..

[37] Foster K (2009). Adeno-Associated Virus-8-Mediated Intravenous Transfer of Myostatin Propeptide Leads to Systemic Functional Improvements of Slow but Not Fast Muscle. Rejuvenation Res..

[38] von Maltzahn J, Chang NC, Bentzinger CF, Rudnicki M (2012). Wnt signaling in myogenesis. Trends Cell Biol.

[39] Jones AE (2015). Wnt/β-catenin controls follistatin signalling to regulate satellite cell myogenic potential. Skelet. Muscle.

[40] Baverstock H, Jeffery NS, Cobb SN (2013). The morphology of the mouse masticatory musculature. J. Anat..

[41] Meng, H. *et al*. Tissue triage and freezing for models of skeletal muscle disease. *J. Vis. Exp*. e51586, 10.3791/51586 (2014). 10.3791/51586 PMC4215994 25078247

[42] Schmittgen TD, Livak KJ (2008). Analyzing real-time PCR data by the comparative C(T) method. Nat. Protoc..

