# Supplementary material for: Wnt7a induces satellite cell expansion, myofiber hyperplasia and hypertrophy in rat craniofacial muscle
Source: Sci Rep. 2018 Jul 13;8:10613. doi: 10.1038/s41598-018-28917-6 (PMC6045621; doi:10.1038/s41598-018-28917-6)
Supplement: Supplementary file 1 — Supplemental files [file 41598_2018_28917_MOESM1_ESM.docx]

Wnt7a induces satellite cell expansion, myofiber hyperplasia and hypertrophy in rat craniofacial muscle

Xu Cheng, Hanyao Huang, Xiangyou Luo, Bing Shi & Jingtao Li*

State Key Laboratory of Oral Diseases & National Clinical Research Centre for Oral Diseases & Department of Oral and Maxillofacial Surgery, West China Hospital of Stomatology, Sichuan University, 14 Ren Min Nan Road, Chengdu, 610041, P. R. China. Correspondence and requests for materials should be addressed to L.J. (email: lijingtao86@163.com)

**Supplemental Table S1.** Animal distribution.

| Observation time points | | 21d | 35d | 56d |
| --- | --- | --- | --- | --- |
| IF | LVP | 6 | 6 | 6 |
|  | MAS | 6 | 6 | 6 |
|  | TA | 6 | 6 | 6 |
| PCR | LVP | 6 | / | / |
|  | MAS | 6 | / | / |
|  | TA | 6 | / | / |
| WB | LVP | 6 | / | / |
|  | MAS | 6 | / | / |
|  | TA | 6 | / | / |

Abbreviations: IF, immunofluorescence; LVP, levator veli palatini; MAS, masseter; TA, tibia anterior.

**Supplemental Table S2.** Antibodies used in this study.

|  | Dilution | Supplier | Catalog number |
| --- | --- | --- | --- |
| Laminin | 1:1000 | Sigma-Aldrich | L9393 |
| Ki67 | 1:500 | Abcam | Ab1667 |
| Pax7 | 1:5 | Developmental Studies Hybridoma Bank | PAX7 |
| MyHC-1 | 1:40 | Developmental Studies Hybridoma Bank | A4.480 |
| MyHC-2A | 1:20 | Developmental Studies Hybridoma Bank | SC-71 |
| MyHC-2X | 1:5 | Developmental Studies Hybridoma Bank | 6H1 |
| MyHC-2B | 1:5 | Developmental Studies Hybridoma Bank | BF-F3 |
| Emb-MyHC | 1:5 | Developmental Studies Hybridoma Bank | BF-G6 |
| Axin2 | 1:1000 | Abcam | ab32197 |
| Frizzled7 | 1:1000 | Abcam | ab64636 |
| Vangl2 | 1:1000 | R&D systems | AF4815 |
| pAkt | 1:1000 | Cell Signaling | #9271 |
| pS6 | 1:1000 | Cell Signaling | #2211 |
| Myostatin | 1:1000 | R&D systems | AF788 |
| Follistatin | 1:1000 | Abcam | ab157471 |

**Supplemental Table S3.** Sequence of primers.

|  | Forward primer (5’-3’) | Reverse primer (5’-3’) |
| --- | --- | --- |
| MyHC-1 | CTTTGATGTGCTGGGCTT | CCTCCCTCTGCTTCTGTTT |
| MyHC-2A | GTGAACTGGAGGGTGAGGT | TCGGTCTTCTTCTGTCTGGT |
| MyHC-2X IIX | CTATCTCTGACAACGCCTACC | GTGCTTCCTCCTTCTTCTTCT |
| MyHC-2B | AAACGAGGTGGAAAATGAAC | TGCTTGTCTCTTGTAGGCTTT |
| myostatin | CAAACAGCCTGAATCCAACTTA | CCGTGAGGGGGTAGCGACAG |
| follistatin | TGGATTAGCCTATGAGGGAAAG | GCCAACCTTGAAATCCCATAG |
| ActRIIB | GGCTCCCTCACGGATTACCT | CATGTACCGTCTGGTGCCAA |

**Supplemental Figure S1.** Quantification of Pax7^+ve^ nuclei (%), Ki67^+ve^ nuclei (%), centrally-nucleated myofiber (%) and myofiber number per field at D21, D35 and D56 after rh-Wnt7a administration.


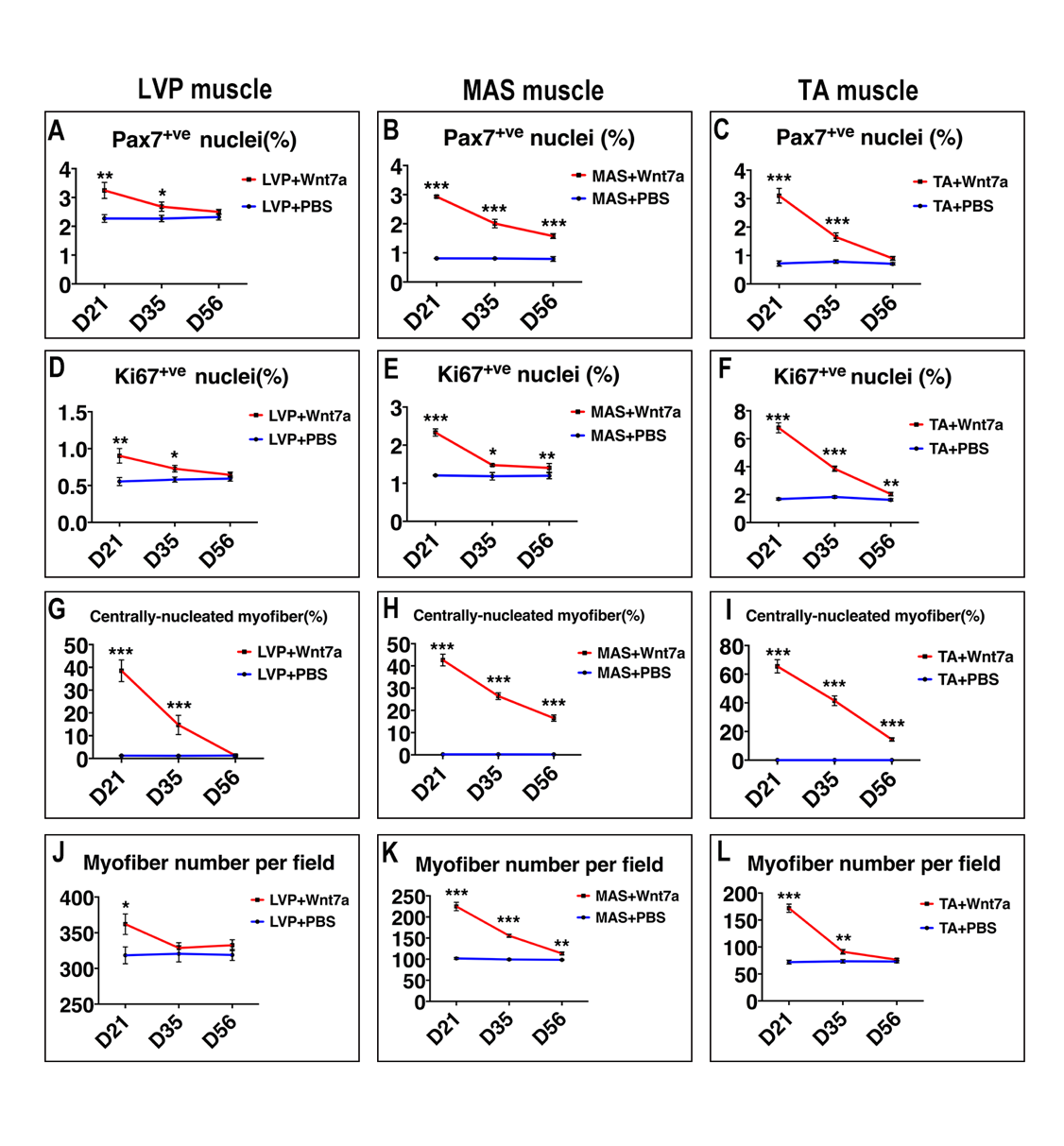


**Supplemental Figure S2.** Scheme of Wnt7a-mediated muscle fiber growth: satellite cell expansion, myofiber hypertrophy and myofiber hyperplasia. Red arrows demonstrate literature data, and blue arrows indicate novel findings about Wnt7a-induced myofiber hyperplasia.


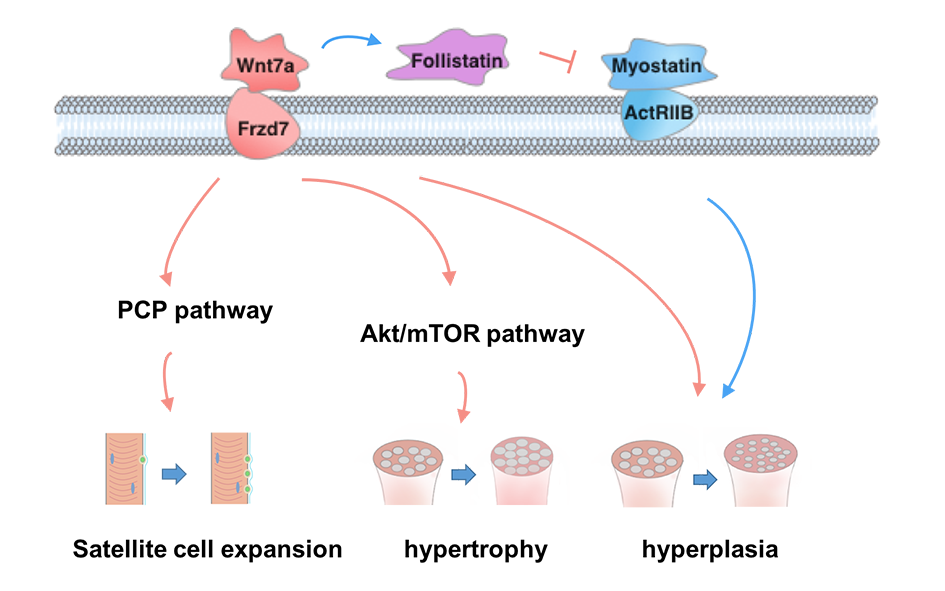


**Supplemental Figure S3.** Full-length gels and blots of Axin2, Frizzled7, Vangl2, Myostatin, Follistatin, pAkt and pS6.


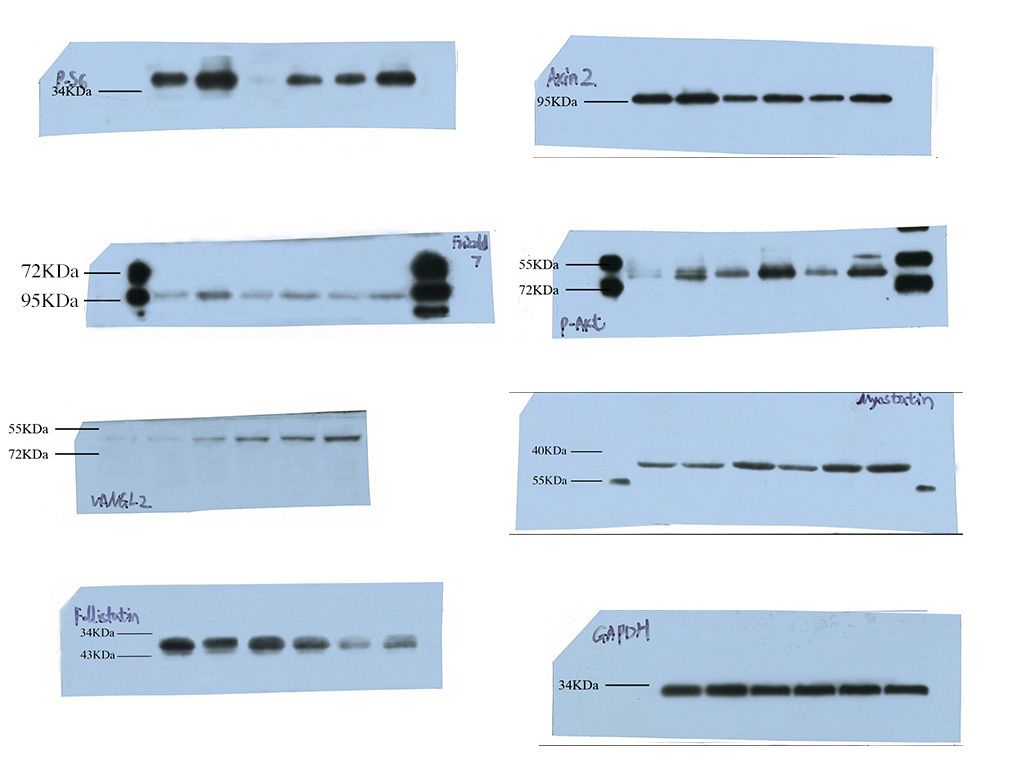


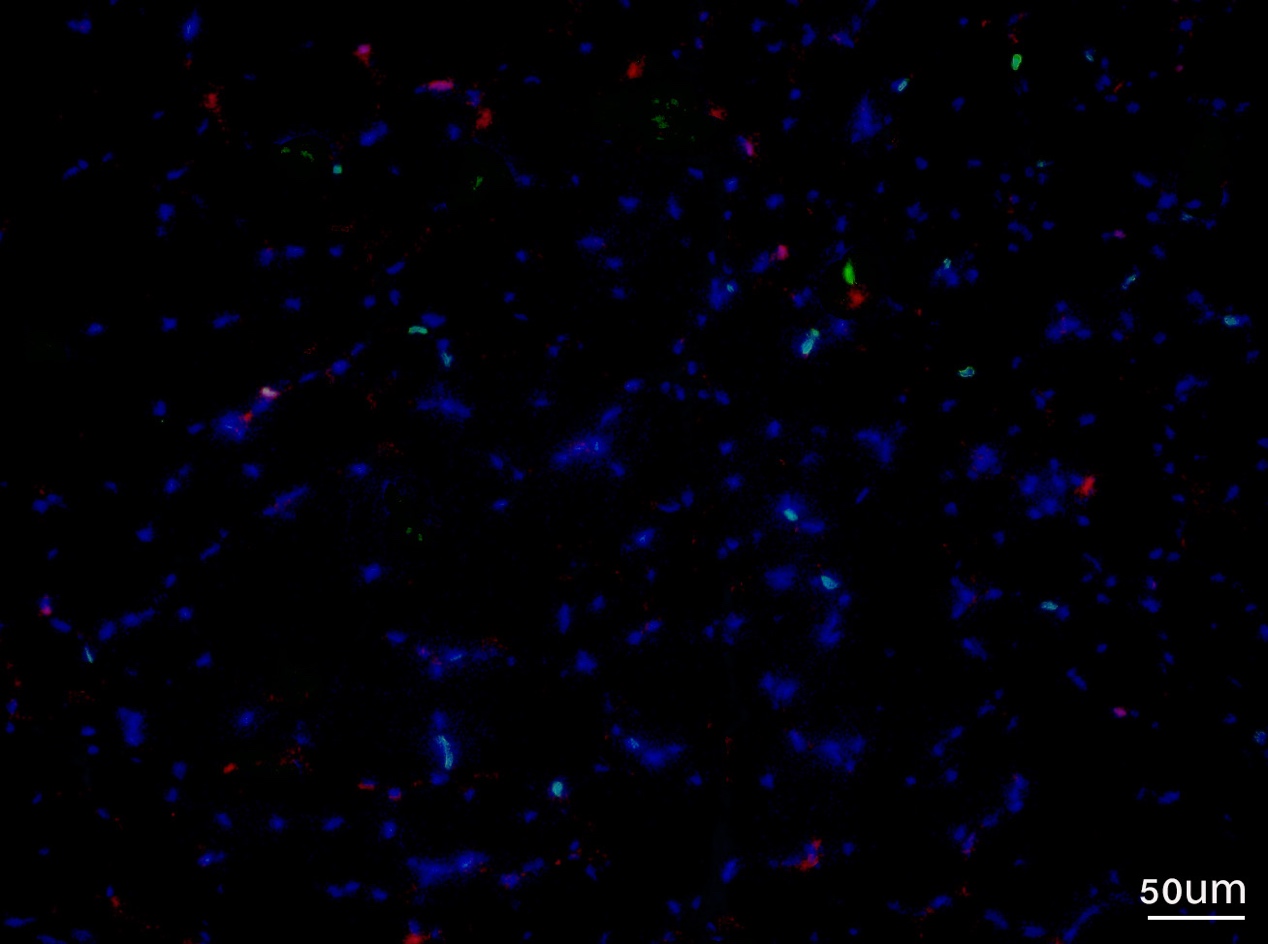


**Supplemental Figure S4.** Immunofluorescence staining of Ki67 (green) and Pax7 (red) and DAPI (blue) in TA muscle at 21 days after rh-Wnt7a administration. Scale bar = 50 um.


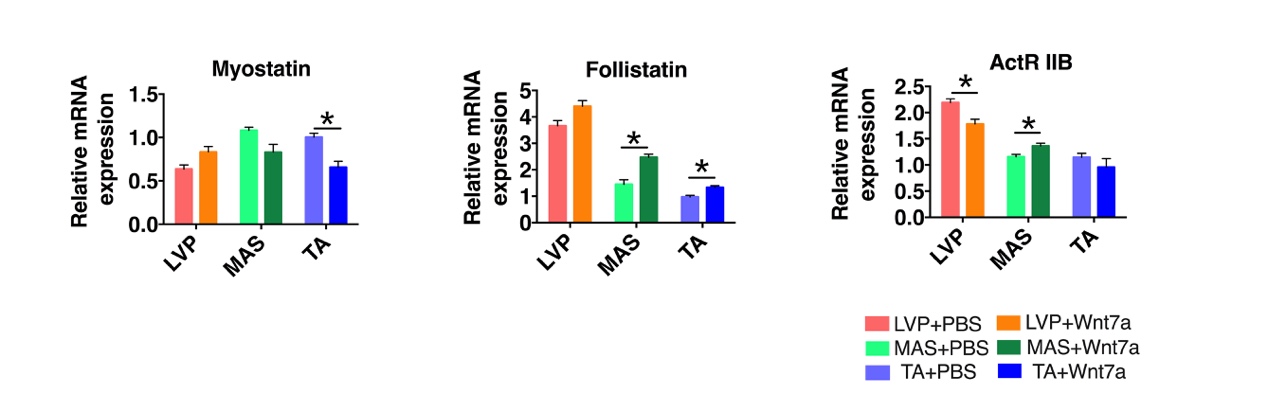


**Supplemental Figure S5.** Relative mRNA expression of myostatin, follistatin and ActR IIB in LVP, MAS and TA muscle in PBS-treated and rh-Wnt7a-treated groups were carried out by quantitative real-time PCR analysis.
